# Is there an influence of perceptual or cognitive impairment on complex sentence processing in hearing aid users?

**DOI:** 10.1371/journal.pone.0291832

**Published:** 2023-09-28

**Authors:** Luise Wagner, Anna-Leoni A. Werle, Antonia Hoffmann, Torsten Rahne, Anja Fengler

**Affiliations:** 1 Department of Otorhinolaryngology, Head and Neck Surgery, University Hospital Halle (Saale), University Medicine Halle (Saale), Halle, Germany; 2 Department of Otorhinolaryngology, Head and Neck Surgery, University Hospital Leipzig, Leipzig, Germany; All India Institute of Speech and Hearing, INDIA

## Abstract

**Background:**

Hearing-impaired listeners often have difficulty understanding complex sentences. It is not clear if perceptual or cognitive deficits have more impact on reduced language processing abilities, and how a hearing aid might compensate for that.

**Methods:**

In a prospective study with 5 hearing aid users and 5 normal hearing, age-matched participants, processing of complex sentences was investigated. Audiometric and working memory tests were performed. Subject- and object-initial sentences from the Oldenburg Corpus of Linguistically and audiologically controlled Sentences (OLACS) were presented to the participants during recording of an electroencephalogram (EEG).

**Results:**

The perceptual difference between object and subject leading sentences does not lead to processing changes whereas the ambiguity in object leading sentences with feminine or neuter articles evokes a P600 potential. For hearing aid users, this P600 has a longer latency compared to normal hearing subjects.

**Conclusion:**

The EEG is a suitable method for investigating differences in complex speech processing for hearing aid users. Longer P600 latencies indicate higher cognitive effort for processing complex sentences in hearing aid users.

## Introduction

With increasing age, people often suffer from hearing loss and a decline in cognitive skills [1, 2]. For people with hearing loss, enormous effort is necessary to discriminate sounds and synthesize speech [3]. More capacity for long-term memory is needed to compensate for difficulties in perception [4]. This compensation requires further cognitive resources which are less available with increasing age [5, 6].

Sentence comprehension crucially relies on identifying who is doing what to whom. To do this, sentence interpretation require sufficient perceptual and cognitive capabilities. Different sentence types and orders of actors in a sentence can challenge these capabilities. In sentences with two potential actors, German speakers prefer to assign the active role (subject) to the first noun phrase (NP) and the passive role (object) to the second one (for further discussion see [7]). Thus, changes in word order increase sentence complexity. Sentences differing from the canonical subject-verb-object (SVO, see Table 1, sentence type 1) word order require the perception and interpretation of linguistic cues such as case marking. In German, case can be marked using masculine articles. However, the differentiation between “der” (NOM ‐ nominative) and “den” (ACC ‐ accusative) relies upon fine-grained perceptual abilities, which are reduced in subjects with hearing loss. In addition, perceptual difficulties can result in increased cognitive processing load, which translates to higher processing costs for non-canonical sentences like object-verb-subject (OVS, see Table 1, sentence type 2) sentences. Ambiguous object-verb-subject sentences (ambOVS, see Table 1, sentence type 3) are cognitively challenging from the start. These sentences contain female (marked by “die”) and neuter nouns (marked by “das”) in the first noun phrase which do not allow for the differentiation of nominative and accusative marked articles. This leads to an ambiguity in object-verb-subjects sentences at the first noun phrase which can be interpreted as both subject and object of the sentences. Only the linguistic information of the second masculine noun phrase allows for the unambiguous interpretation. Thus information of the first noun phrase needs to be stored in the working memory until the disambiguating information can be processed. According sentence structures are found in the Oldenburg Corpus of Linguistically and Audiologically controlled Sentences (OLACS, [8], which was used in this study (examples see Table 1).

**Table 1:** Blue indicates nominative subject and green the accusative object of the sentence. In German the articles give hints of the case. In the female case of the duck the NP2 with the dog needs to be reached to find out the actor. | marks the position of the triggers.

	Sentence Type	Example in German and English
1	SVO (subject–verb–***object***) NP1 (NOM)–verb–***NP2 (ACC)***	|Der dicke Bär interviewt |***den*** kleinen Pinguin.
		The thick bear interviews the little penguin.
2	OVS (***male object***–verb–subject) ***NP1 (ACC)***–verb–NP2 (NOM)	|***Den*** kleinen Pinguin interviewt |der dicke Bär.
		The little penguin is interviewed by the thick bear.
3	ambOVS (***feminine/neuter object***–verb–subject) ***NP1 (ACC)***–verb–NP2 (NOM)	|***Die*** nasse Ente tadelt |der treue Hund
		The wet duck is criticized by the trusty dog.

Electroencephalography (EEG) is a commonly used tool for investigating language processing. With EEG one can measure event related potentials (ERPs) by presenting visually or acoustically stimulating triggers. There are different acoustically in sentence processing evoked potential types e.g. the P600 component [9, 10], reflecting aspects of combinatorial processing, e.g. the resolution of syntactic errors [11, 12] respectively integrative or interpretative brain processes [13–16] which are associated with higher memory costs; or the phenomenon of the early left anterior negativity (ELAN) that is linked to increasing effort and syntactic integration difficulties [17, 18]. Both potentials are indicators of a higher impact on working memory [19, 20]. In subjects with hearing impairment, perceptual difficulties during integration of sentence parts into the context are very likely to occur, and therefore ELAN and P600 as associated potentials are expected [21]. Additionally, cognitive difficulties would be reflected by P600 and LAN [22].

Studies with hearing-impaired participants show a slowdown in processing. For object-initial sentences, the responses are delayed [23], and Wendt et al. [24] showed this with eye tracking experiments using OLAC sentence material as used in our study. With the same sentence materials, MRI studies have shown that there is in increased activity in the left superior frontal gyrus in hearing-impaired individuals as compared to normal hearing peers when processing complex sentences. Further the same study proved that this changes with hearing aid use and more cortical regions outside the temporal cortex will be activated [25].

The aim of the study was to investigate whether and how perceptual deficits in hearing-impaired subjects lead to higher cognitive load during complex sentence processing. To do this, an EEG was recorded while listening to SVO, OVS and ambOVS sentences. We hypothesise that perceptual deficits result in processing difficulties in the hearing-impaired group, which will be reflected by an increased ELAN component at the article of the second noun phrase in OVS sentences. Following reanalysis should be reflected by a following P600. Further, we expect a P600 component in ambiguous OVS sentences due to higher cognitive effort. Psychoacoustic phoneme discrimination tests as well as working memory tests were introduced to assess reasons for changes in processing effort. We infer that perceptual difficulties would result in different cortical potentials for object leading compared to subject leading sentences and cognitive deficits would have a larger impact on the ERPs for the ambiguous sentences. Furthermore, if we can show that EEG with these sentence types is an appropriate method for measuring perceptual differences, further studies with cochlear implant patients who are not suitable for MRI studies, can give insight into speech recognition processes in this patient group.

## Material and methods

### Subjects

In an exploratory case-control study from December 2021 until November 2022, participants were acquired through personal contact and information on bulletin boards. All data are pseudonymized and just authors can reconstruct by a key list the suitable participants. Inclusion criteria were an age of more than 18 years and German as the mother tongue. The level of education and handedness were determined in an interview. The participants were divided into hearing aid group and an age-matched (± 2 years) normal hearing group.

In the normal hearing group a pure-tone hearing threshold (4PTA, average at 0.5, 1, 2, and 4 kHz) of better than 20 dB was required. The hearing aid group had to be binaurally aided, for at least 6 months, with a stable fitting. There are no restrictions on hearing aid type. The aided 4PTA in open sound field needs to be better than 40 dB and a minimal monosyllable aided word recognition score of 70% at 65 dB SPL was required as well.

To exclude subjects with undetected severe cognitive deficits, all participants had to complete the Montreal Cognitive Assessment (MoCA, version 7 (2004) from https://mocacognition.com/) and pass it with normal scores, i.e., at least 26 out of 30 points [26]. Further exclusion criteria were alcohol abuse, depression, tinnitus, dementia, amblyopia, dyslexia, or medication with neural effect, e.g., antidepressiva, as assessed by questionnaire.

Written informed consent was obtained from all subjects prior to inclusion in the study. The study protocol is in line with the Declaration of Helsinki and the local ethics conventions (ethics committee of the Martin Luther University of Halle-Wittenberg; approval number: 2020–119).

#### Psychoacoustic and cognitive assessments

To evaluate the perceptual skills, pure tone audiometry and the German Freiburger Monosyllabic tests as well as the WAKO (rhymes developed by Wallenberg and Kollmeier) [27, 28] was performed on the participants in a sound- attenuated booth (Industrial Acoustics Company, Niederkrüchten, Germany) with an AT1000 clinical audiometer (Auritec, Hamburg, Germany) and loudspeakers in 0° azimuth with a distance of 1 m in free field condition. For pure tone audiometry HDA300 headphones (Sennheiser electronic GmbH & Co.KG, Wedemark, Germany) were used. The WAKO was used to test the phoneme discrimination abilities.

Cognitive capabilities were determined by a visual span as well as an n-back task [29]. Both tasks rely on working memory capacities. Visual presentation was chosen to minimize the impact of perceptual difficulties on the task. The visual span was tested using a PsychoPy script (Standalone PsychoPy 2021.2.3). In a digit span test, the participants had to memorise numerical series that were presented on a screen, each digit for one second. The number of digits presented increased after two correct repetitions of the series, and the test ended after two successive wrong answers. The highest number of correct repetitions was used as an indicator of visual span capabilities. In the n-back task, squares were visually presented in a 3x3 matrix for 1.25 s with a following gap of 1.25 s at different positions on a screen. The participants were required to recall these. If a square appeared at the same position as “n” positions before the participant had to indicate this by pressing a button. A 1-back test was performed as training for the 2-back test. The correct percentage of answers to the 2-back test was used for the evaluation of working memory.

For analysis *t*-test are used to compare the scores of normal hearing participants and hearing aid users for the Freiburger Monosyllabic test, the WAKO, the MoCa, the digit-span ‐ and the 2-back test.

### Stimuli

For the following EEG measurement, sentences from the Oldenburg Corpus of Linguistically and Audiologically controlled Sentences (OLACS, [8]) were used. All sentences contain seven words with 11–13 syllables, describing a human or animal doing something with a ’human-like’ animal or vice versa. Pairs of pictures illustrating the sentences with swapped actors were presented as well. The sentence configurations used were subject-verb-object (SVO), object-verb-subject (OVS), and ambiguous object-verb-subject sentences (ambOVS) (see Table 1).

All sentences consisted of two noun phrases (NP1 and NP2) and one verb. In German, overt case-marking appears only in masculine articles. In subject noun phrases, the nominative case (NOM) is marked by “der”, in object noun phrases the accusative (ACC) is marked by “den”. Thus, SVO sentences follow the structure of NP1 (NOM)–Verb–NP2 (ACC), OVS sentences follow the structure NP1 (ACC)–Verb–NP2 (NOM). Therefore, the relevant morphological cue that leads to differences in interpretation is marked in NP1.

In contrast, in ambOVS sentences, the first noun phrase is ambiguous due to the feminine or neuter article. Only the second noun phrase provides the crucial information (NOM) necessary for the correct interpretation. However, due to the preferred interpretation of NP1 as a subject, the sentence interpretation needs to be overwritten after disambiguating information in NP2. This is challenging for the working memory.

Thirty sound files per sentence type were used. For each sentence, a picture and one picture with exchanged actors exists. These two were presented simultaneously next to each other on a screen, with the participant having to select the correct one. Each sentence was presented twice, once with the correct picture on the right, once on the left side. A total of 180 sentences were presented in pseudorandomised order, divided into 18 blocks of 10 sentences each. The participant had to decide which picture corresponds to the sentence and press a response button (right with the right thumb, left with the left thumb).

Stimulus presentation was controlled by STIM2 software (Compumedics Neuroscan, Singen, Germany). Two triggers were set, the first on the beginning of the whole sentence, the second on the article of the NP2. Responses were recorded after sentence presentation.

### Electrophysiology

In a sound-isolated room, the participants sat on a comfortable armchair. The visual presentation was realized with a screen at 1 m distance. The acoustic stimuli were presented by loudspeakers at a distance of 1 m with a sound pressure level of 70 dB SPL calibrated using a sound level meter (Type 2250, Brüel & Kjær, Naerum, The Netherlands).

Stimulation and acquisition were achieved by a system consisting of a stimulation computer with STIM2 software (Compumedics Neuroscan, Singen, Germany), a StimTrackerDuo (Cedrus Corporation, San Pedro, CA, USA), a SynAMPs RT (Compumedics Neuroscan, Singen, Germany) and an EasyCap (EASYCAP GmbH, Woerthsee-Etterschlag, Germany). The triggers were sent by STIM2 and the StimTrackerDuo to the recording computer on which the Curry 7 software (Compumedics Neuroscan, Singen, Germany) recorded the data. Participants responded using the RB-7XX response pad (Cedrus Corporation, San Pedro, CA, USA).

Sixty-four electrodes were gelled on a standard EEG cap (EASYCAP GmbH, Herrsching, Germany) according to the international 10-20-system [30], with one being a nose electrode as a possible reference. Hardware filters were set to 500 Hz low pass and a sampling frequency of 1 kHz was used. Before recording, the impedances at the electrodes were ensured to be homogeneous and around 10 kOhm. They were tested and documented before and after the whole recording.

### Data processing

The recorded EEG of all blocks was exported in December 2022 from Curry software (Compumedics Neuroscan, Singen, Germany) as .ceo (events), .dap (data parameter), .rs3 (geometry) and.dat (data) files and imported to Python with the MNE toolbox (version 1.2). The data were then concatenated to one continuous dataset. A bandpass filter with a Hamming window of 1 to 30 Hz was applied. Subsequently, bad channels were removed when they where without signal or with very noisy signal over a longer time. Beside visual identification in the continuous data and the power spectral density was examined (Welch’s method [31]) further channels were removed if after the independent component analysis (ICA) if they were effecting more than 3 channels in an unsual way. A first epoching was done. The data were segmented in epochs, starting 1 s before and ending 1.5 s after each event trigger. The sentences were then grouped according to their trigger codes into SVO, OVS, and ambOVS sentences. Response triggers were analysed and all sentences with wrong responses were identified and excluded from analysis. To reduce artefacts an ICA was used. The ICA components were calculated from the previously formed epochs, with the most common artefacts being from eye movements. After visual inspection, the selected components were removed from the continuous data.

The two triggers included in the recordings of all sentences were used for final analysis epoching. Therefore, epochs from 0 to 1.5 s were calculated starting at each trigger, including a baseline correction with the data from 1 to 0 s regarding the first trigger of the sentence. All epochs of each trigger and sentence-type were averaged resulting in six ERP curves per subject and recording electrode. For each response of the first sentence part (NP1) the maximal and minimal amplitudes and their corresponding latency at POz were determined; for the second part the maximal ones were determined. Therefore, the minimum and maximum in a range from 0 to 1000 ms were determined, and the corresponding latencies were registered. A grand average was calculated for both participant groups, and plotted.

In the averages following the second trigger, the significance of the ERP response (maxima) was tested using a *t*-test. In case of a significance a topoplot for a time window ± 20 ms around this maximum was generated.

## Results

Five hearing aid users (mean age (58.2 ± 14.0) years, 3 female, 1 left handed) and five age-matched (± 2 years)) normal hearing participants (mean age (58.2 ± 13.1) years, 4 female, 1 left handed) took part in the study.

The aided PTA4 for the hearing aid group in open sound field was (27 ± 8) dB HL. In the normal hearing control group headphones were used and mean thresholds of (16 ± 10) dB HL for the right ear and (16 ± 11) dB HL for the left ear were measured. The mean WRS for monosyllables at 65 dB SPL was (93 ± 10)% in the hearing aid group and (96 ± 10)% in the control group (*t*(8) = 0.67, *p* > 0.05). The mean WAKO phoneme test score was (24.4. ± 0.9) for hearing aid users and (24.2 ± 1.1) out of 25 for normal hearing participants (*t*(8) = -0.31, *p* > 0.05).

The average score in the MOCA test was (28.8 ± 1.1) for hearing aid users and (28.6 ± 1.9) for the control group and thus in the normal range (>26). The scores were not significantly different (t(8) = -0.20, *p* > 0.05). In the digit span test the hearing aid group remembered on average (5.4 ± 0.9) digits and the normal hearing control group (5.4 ± 0.5) digits (*t*(8) = 0.0, *p* = 0.5). In the 2-back test, hearing aid users had (94 ± 4) % correct answers and normal hearing control (93 ± 10) %. Both test results were not different between the groups (*t*(8) = -0.14, *p* > 0.05). The educational background was also comparable between the groups, since three hearing aid users and two participants in the control group achieved a university degree.

**Fig 1:**
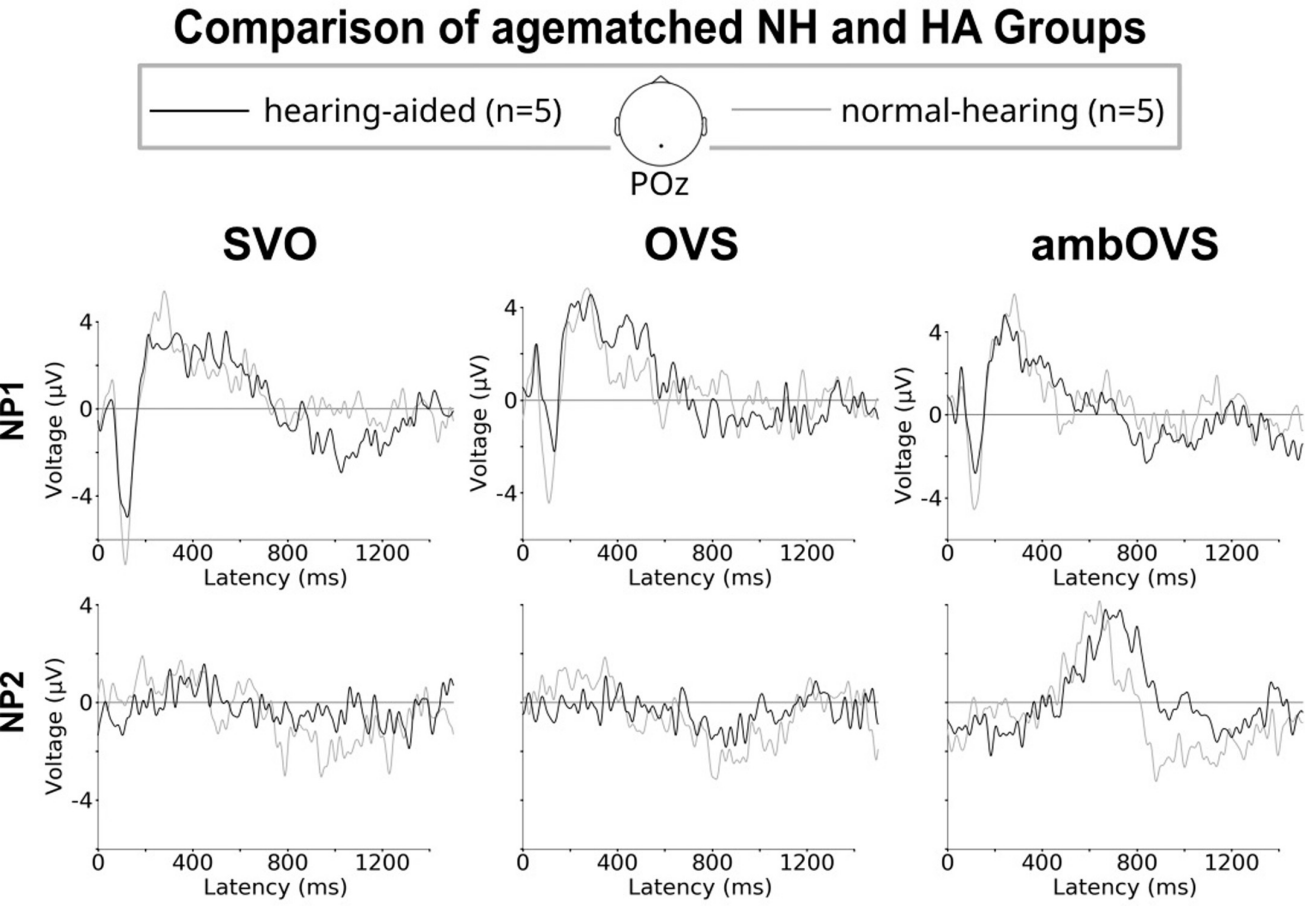
Grand average waveforms for all correctly answered sentences at POz for the hearing aid group (black) and the normal hearing control (grey). The upper row shows the potentials after the beginning of the sentence (NP1), the lower row the potentials after the first word of the second sentence part (NP2). Columns show the different sentence types (SVO: NP1 (NOM)–verb–NP2 (ACC), OVS: NP1 (ACC)–verb–NP2 (NOM), ambOVS: NP1 (ACC)–verb–NP2 (NOM)). NP1 sentences evoke a clear P1-N1-P2 waveform. A P600 response was evoked in the second part of the ambOVS sentences with shorter latencies for normal hearing listeners.

The grand average ERP responses are shown in Fig 1. The first sentence part (NP1, first trigger) evoked a clear P1-N1-P2 complex for all participants (first row). Only sentences with correct answers were analysed. Hearing aid users reached (97 ± 1)% correct answers during the EEG experiment and normal hearing controls (98 ± 3)%.

The second row in Fig 1 shows the waveform following the second trigger (NP2), that is, the second part of the sentence (NP2) in which the listener finally learns who is the actor, who is doing what with whom. For SVO and OVS sentences no significant evoked responses are seen. For the ambOVS sentences a significant response is seen at a latency of about 600 ms and interpreted as P600. The amplitudes were (4.03 ± 0.41) μV for hearing aid users and (4.28 ± 0.61) μV for normal hearing listeners. The difference was not significant (*t*(8) = -0.75, *p* > 0.05). The P600 latencies were (720 ± 54) ms for hearing aid users and significantly shorter for normal hearing listeners (631 ± 27) ms, *t*(8) = -3.34, *p* < 0.01).

**Fig 2:**
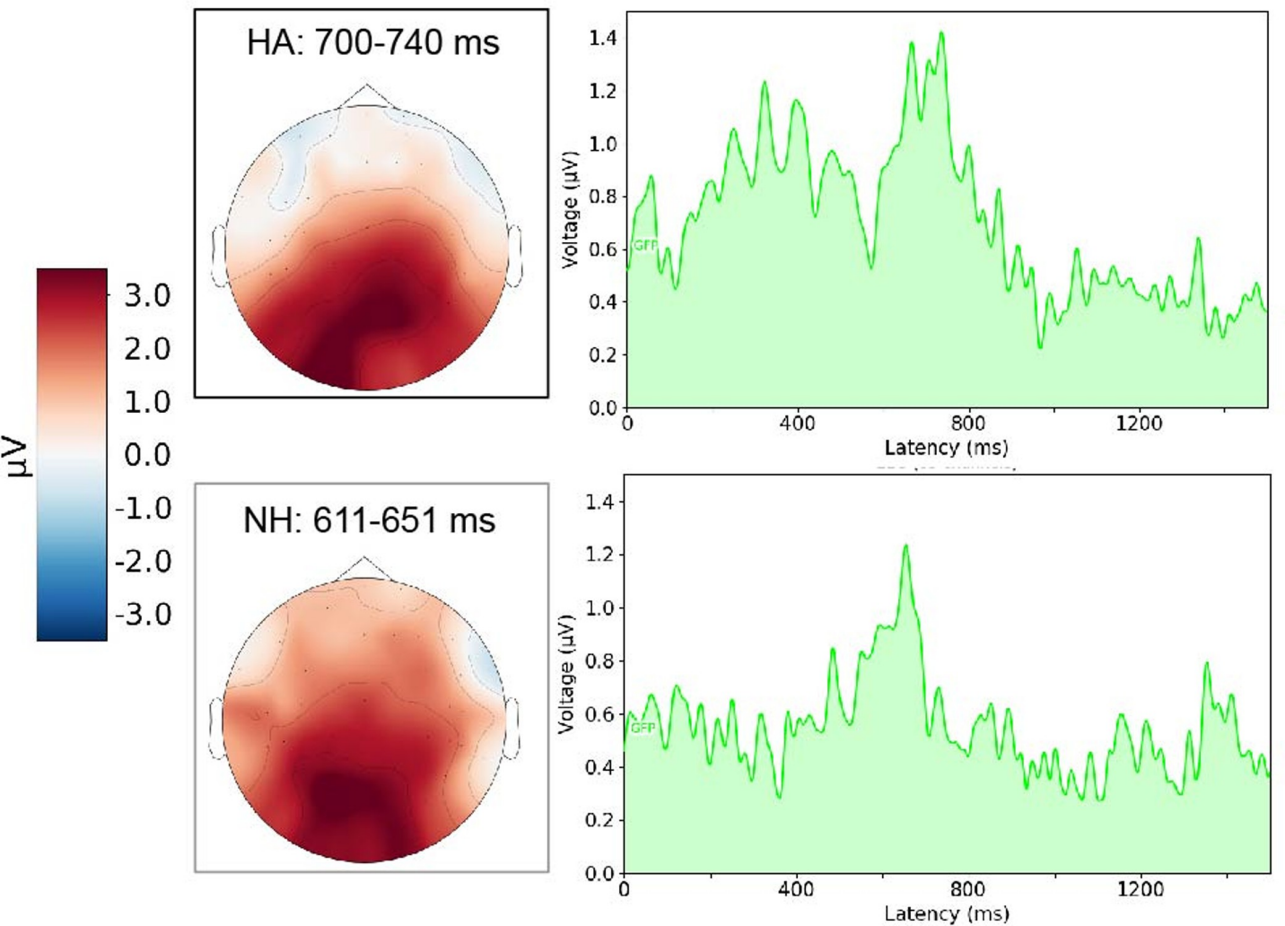
Mean global field power for the second part of the ambiguous OVS sentences (NP2). The topoplots show a centro parietal positivity in a time window 40 ms around the P600 maximum.

Fig 2 shows the mean global field power of NP2 and topoplots with a positive centro parietal activity in a time window 40 ms around the amplitude maximum for the second part of the ambiguous OVS sentences, i.e., the P600.

## Discussion

The main objective of the study was to investigate whether and how perceptual deficits in subjects with hearing impairment lead to a higher cognitive load during complex sentence processing compared to normal hearing listeners. In both groups, no processing difficulties could be detected for OVS sentences compared to SVO sentences. Following these findings, the hypothesis that perceptual deficits in subjects with hearing aids may lead to difficulties in processing the article information cannot be confirmed. Therefore, we conclude that in our group the perceptual deficits were not large enough to have an impact on processing or, in other words, were sufficiently restored by the hearing aids used. The results of the psychoacoustic tests confirm this argument since the achieved scores were comparable. Word and phoneme recognition scores were not different between the groups. The difference in pure-tone hearing thresholds observed obviously did not contribute in a relevant way to a different perception of speech and listening effort.

However, despite comparable cognitive capabilities, we could detect differences in processing ambiguous OVS between groups. In both groups, a P600 could be detected at the article of the second noun phrase, which indicates a more difficult analysis of these sentences by activating more cognitive resources. This is in line with other studies showing that an increase in cognitive load is reflected by a P600 component [10, 17]. In the hearing aid user group, a later P600 latency was measured which reflects a slowing of processing speed [32]. However, comparable numbers of correct answers indicate no difference in sentence comprehension between groups. Further studies are required to evaluate whether the slowing of processing speed may result in faster hearing fatigue and may explain higher listening effort in hearing aid users (for a review see Ohlenforst et al. [33]). One has to consider the technical delay in hearing aids, but this is with about 10 ms much shorter than the latency shift of the P600 observed in this study, so there shouldn’t be an effect.

This study shows that differences in sentence processing between participants with normal hearing and hearing-impaired participants can be assessed with EEG. The results are consistent with results of eyetracking experiments by Wendt et al. showing that increasing complexity leads to higher processing effort [34]. Furthermore, it is consistent with the results of fMRI experiments with OLACs material, indicating that hearing loss affects language processing but mainly in complex and not in simple sentences [24, 25].

The novelty of this study is that differences in complex sentence processing can also be seen in EEG recordings in hearing aid users. Hearing aid sound processing did not distort the acoustic signal to such an extent that significant differences would have occurred at the perceptual level but we could show in our participants that even with the same cognitive abilities the hearing impaired participants have a higher cognitive effort in processing more complex sentences. Nevertheless, further investigation is needed, especially with cochlear implant users who are not able to participate in functional MRI experiments since the audio processors do not work inside the scanner.

In further studies, an extension of sentence material would help to distinguish more detailed between perceptual and cognitive sources of impairment. Gardenpath sentences [35], e.g., would increase the complexity of the sentences and enable the investigator to have a wider range to differentiate the results. If participants with different degrees of hearing impairment, or even users of cochlear implants, were included in such studies, more information could be gained on the influence of the perceptual component on complex sentence processing. Therefore, further studies with varying degrees of hearing impairment and a larger set of sentences could help in learning more about the role of cognitive or perceptual deficits and develop different strategies to overcome them.

## Supporting information

S1 File Individual scores for all tests for each participant. (PDF)

S2 File Individual curves for the second part of the ambiguous OVS sentences for each participant. (PDF)

## References

[1] Schneider BA, Pichora-Fuller MK, Daneman M. Effects of senescent changes in audition and cognition on spoken language comprehension. In: Gordon-Salant S, editor. The aging auditory system. New York, NY: Springer; 2010. pp. 167–210.

[2] Pichora-Fuller MK. Cognitive aging and auditory information processing. Int J Audiol. 2003; 42:26–32. doi: 10.3109/14992020309074641 12918626

[3] Plomp R. Auditory handicap of hearing impairment and the limited benefit of hearing aids. J Acoust Soc Am. 1978; 63:533–49. doi: 10.1121/1.381753 670550

[4] Rönnberg J. Cognition in the hearing impaired and deaf as a bridge between signal and dialogue: a framework and a model. Int J Audiol. 2003; 42:68–76. doi: 10.3109/14992020309074626 12918612

[5] Rönnberg J, Rudner M, Foo C, Lunner T. Cognition counts: a working memory system for ease of language understanding (ELU). Int J Audiol. 2008; 47 Suppl 2:S99–105. doi: 10.1080/14992020802301167 . 19012117

[6] Slade K, Plack CJ, Nuttall HE. The Effects of Age-Related Hearing Loss on the Brain and Cognitive Function. Trends Neurosci. 2020; 43:810–21. Epub 2020/08/19. doi: 10.1016/j.tins.2020.07.005 . 32826080

[7] Primus B. Cases and thematic roles: ergative, accusative and active. Tübingen: Niemeyer Verlag; 1999.

[8] Uslar VN, Carroll R, Hanke M, Hamann C, Ruigendijk E, Brand T, et al. Development and evaluation of a linguistically and audiologically controlled sentence intelligibility test. J Acoust Soc Am. 2013; 134:3039–56. doi: 10.1121/1.4818760 . 24116439

[9] Leckey M, Federmeier KD. The P3b and P600(s): Positive contributions to language comprehension. Psychophysiology. 2020; 57:e13351. Epub 2019/02/25. doi: 10.1111/psyp.13351 . 30802979 PMC7934419

[10] Gouvea AC, Phillips C, Kazanina N, Poeppel D. The linguistic processes underlying the P600. Lang Cognitive Proc. 2010; 25:149–88. doi: 10.1080/01690960902965951

[11] Osterhout L, Holcomb PJ. Event-related brain potentials elicited by syntactic anomaly. J Mem Lang. 1992; 31:785–806. doi: 10.1016/0749-596X(92)90039-Z

[12] Hagoort P, Brown C, Groothusen J. The syntactic positive shift (sps) as an erp measure of syntactic processing. Lang Cognitive Proc. 1993; 8:439–83. doi: 10.1080/01690969308407585

[13] Brouwer H, Fitz H, Hoeks J. Getting real about semantic illusions: rethinking the functional role of the P600 in language comprehension. Brain Res. 2012; 1446:127–43. Epub 2012/02/02. doi: 10.1016/j.brainres.2012.01.055 . 22361114

[14] Friederici AD. The brain basis of language processing: from structure to function. Physiol Rev. 2011; 91:1357–92. doi: 10.1152/physrev.00006.2011. . 22013214

[15] Kaan E. Event-Related Potentials and Language Processing: A Brief Overview. Lang Linguist Compass. 2007; 1:571–91. doi: 10.1111/j.1749-818X.2007.00037.x

[16] Osterhout L. A Superficial Resemblance Does Not Necessarily Mean You Are Part of the Family: Counterarguments to Coulson, King and Kutas (1998) in the P600/SPS-P300 Debate. Lang Cognitive Proc. 1999; 14:1–14. doi: 10.1080/016909699386356

[17] Kaan E, Harris A, Gibson E, Holcomb P. The P600 as an index of syntactic integration difficulty. Lang Cognitive Proc. 2000; 15:159–201. doi: 10.1080/016909600386084

[18] Hagoort P, Brown CM. ERP effects of listening to speech compared to reading: the P600/SPS to syntactic violations in spoken sentences and rapid serial visual presentation. Neuropsychologia. 2000; 38:1531–49. doi: 10.1016/s0028-3932(00)00053-1 10906378

[19] Münte TF, Schiltz K, Kutas M. When temporal terms belie conceptual order. Nature. 1998; 395:71–3. doi: 10.1038/25731 . 9738499

[20] Osterhout L, Holcomb PJ, Swinney DA. Brain potentials elicited by garden-path sentences: Evidence of the application of verb information during parsing. Journal of Experimental Psychology: Learning, Memory, and Cognition. 1994; 20:786–803. doi: 10.1037//0278-7393.20.4.786 8064247

[21] Friederici AD. Towards a neural basis of auditory sentence processing. Trends in Cognitive Sciences. 2002; 6:78–84. doi: 10.1016/s1364-6613(00)01839-8 15866191

[22] Meltzer JA, Braun AR. P600-like positivity and Left Anterior Negativity responses are elicited by semantic reversibility in nonanomalous sentences. J Neurolinguistics. 2013; 26. doi: 10.1016/j.jneuroling.2012.06.001 . 24227906 PMC3822000

[23] Tun PA, Benichov J, Wingfield A. Response latencies in auditory sentence comprehension: effects of linguistic versus perceptual challenge. Psychol Aging. 2010; 25:730–5. doi: 10.1037/a0019300 . 20853977 PMC3020665

[24] Wendt D, Kollmeier B, Brand T. How hearing impairment affects sentence comprehension: using eye fixations to investigate the duration of speech processing. TRENDS IN HEARING. 2015; 19. doi: 10.1177/2331216515584149 . 25910503 PMC4409940

[25] Vogelzang M, Thiel CM, Rosemann S, Rieger JW, Ruigendijk E. Effects of age-related hearing loss and hearing aid experience on sentence processing. Sci Rep. 2021; 11:5994. Epub 2021/03/16. doi: 10.1038/s41598-021-85349-5 . 33727628 PMC7971046

[26] Nasreddine ZS, Phillips NA, Bédirian V, Charbonneau S, Whitehead V, Collin I, et al. The Montreal Cognitive Assessment, MoCA: a brief screening tool for mild cognitive impairment. J Am Geriatr Soc. 2005; 53:695–9. doi: 10.1111/j.1532-5415.2005.53221.x . 15817019

[27] Sotscheck J. Ein Reimtest für Verständlichkeitsmessungen mit deutscher Sprache als ein verbessertes Verfahren zur Bestimmung. Dissertation, Technische Hochschule. 1982.

[28] von Wallenberg E-L, B Kollmeier. Sprachverständlichkeitsmessungen für die Audiologie mit einem Reimtest in deutscher Sprache. Erstellung un Evaluation von Testlisten. Audiologische Akustik. 1989; 28:50–6.

[29] Jaeggi SM, Buschkuehl M, Perrig WJ, Meier B. The concurrent validity of the N-back task as a working memory measure. Memory. 2010; 18:394–412. Epub 2010/04/19. doi: 10.1080/09658211003702171 . 20408039

[30] Jasper. Report of the committee on methods of clinical examination in electroencephalography. Electroen Clin Neuro. 1958; 10:370–5. doi: 10.1016/0013-4694(58)90053-1

[31] Welch P. The use of fast Fourier transform for the estimation of power spectra: A method based on time averaging over short, modified periodograms. IEEE Trans Audio Electroacoust. 1967; 15:70–3. doi: 10.1109/TAU.1967.1161901

[32] Yumba WK. Cognitive Processing Speed, Working Memory, and the Intelligibility of Hearing Aid-Processed Speech in Persons with Hearing Impairment. Front Psychol. 2017; 8:1308. Epub 2017/08/15. doi: 10.3389/fpsyg.2017.01308 . 28861009 PMC5559705

[33] Ohlenforst B, Zekveld AA, Jansma EP, Wang Y, Naylor G, Lorens A, et al. Effects of Hearing Impairment and Hearing Aid Amplification on Listening Effort: A Systematic Review. Ear Hear. 2017; 38:267–81. doi: 10.1097/AUD.0000000000000396 . 28234670 PMC5405775

[34] Wendt DC, Brand T, Kollmeier B. An eye-tracking paradigm for analyzing the processing time of sentences with different linguistic complexities. 2014. Available from: 10.1371/journal.pone.0100186. PMC4065036 24950184

[35] Ferreira F, Henderson JM. Recovery from misanalyses of garden-path sentences. J Mem Lang. 1991; 30:725–45. doi: 10.1016/0749-596X(91)90034-H

