# Supplementary material for: Is there an influence of perceptual or cognitive impairment on complex sentence processing in hearing aid users?
Source: PLoS One. 2023 Sep 28;18(9):e0291832. doi: 10.1371/journal.pone.0291832 (PMC10538791; doi:10.1371/journal.pone.0291832)
Supplement: S1 File — (PDF) [file pone.0291832.s001.pdf]

ID	age	gender	handedness	hearing	Freiburger monosyllables	WAKO	Moca	maximal digit-span	2-back	percentage correct during EEG paradigm
102	53	f	R	NH	100%	25	30	6	100%	99.44%
105	45	f	R	NH	100%	25	30	6	93%	98.33%
106	50	m	L	NH	95%	25	30	5	100%	99.44%
110	77	f	R	NH	85%	23	27	5	97%	92.78%
112	66	f	R	NH	100%	23	26	5	77%	98.33%
201	44	f	L	HA	100%	24	28	5	90%	97.78%
202	65	m	R	HA	85%	25	30	6	100%	98.89%
203	49	f	R	HA	100%	25	28	4	93%	95.56%
206	54	f	R	HA	95%	25	30	6	90%	97.78%
207	79	m	R	HA	85%	23	28	6	97%	96.11%

f…female
m… male
R… right handed
L… left handed
HA… hearing aid
NH… normal hearing
