# Supplementary figures and images for: Is there an influence of perceptual or cognitive impairment on complex sentence processing in hearing aid users?
Source: PLoS One. 2023 Sep 28;18(9):e0291832. doi: 10.1371/journal.pone.0291832 (PMC10538791; doi:10.1371/journal.pone.0291832)

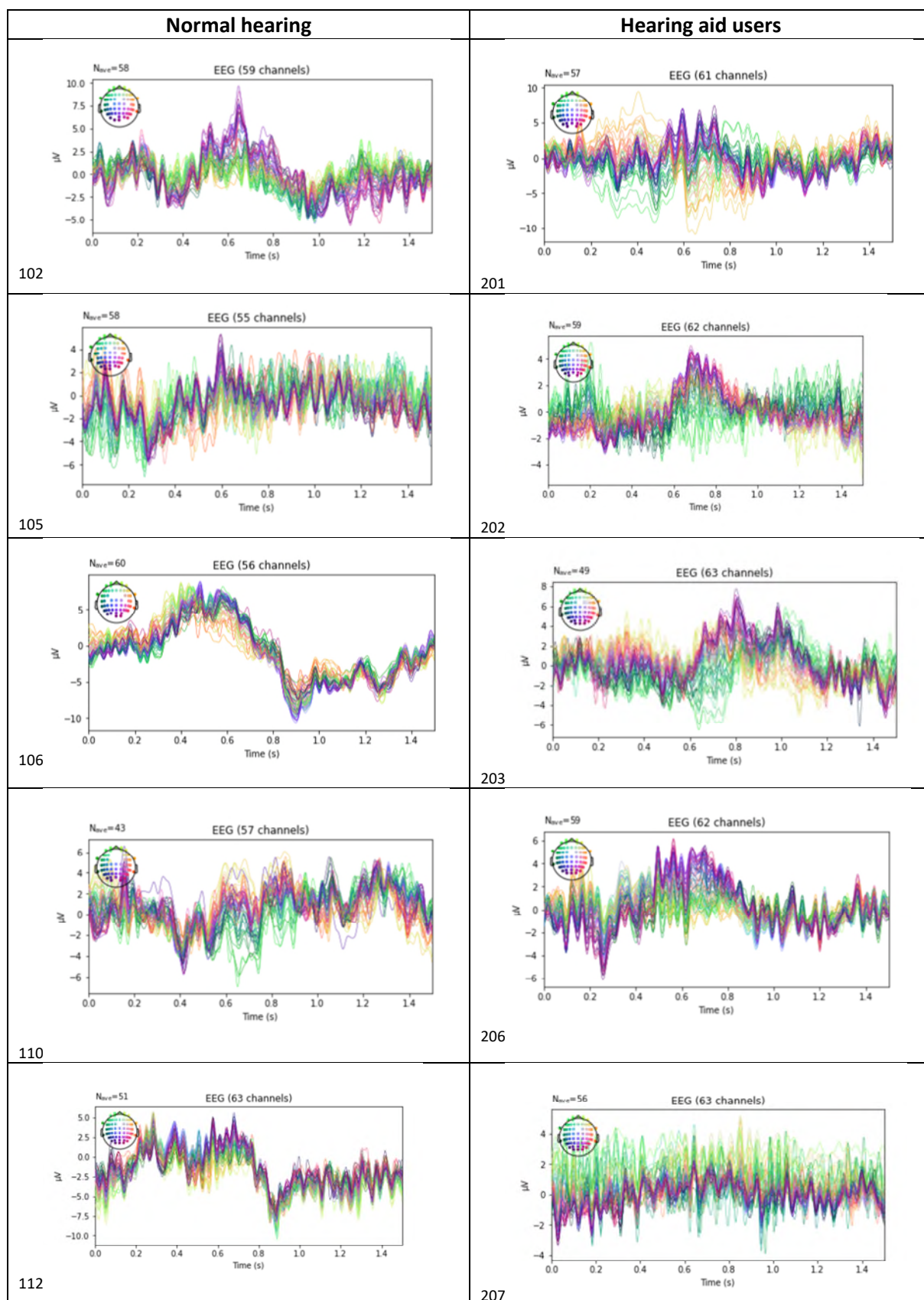

Supplement: S2 File — (PDF) [file pone.0291832.s002.pdf]
